# Defining the role of Interleukin-6 for the development of perioperative neurocognitive disorders: Evidence from clinical and preclinical studies

**DOI:** 10.3389/fnagi.2022.1097606

**Published:** 2023-01-26

**Authors:** Odmara L. Barreto Chang, Mervyn Maze

**Affiliations:** ^1^Department of Anesthesia and Perioperative Care, University of California, San Francisco, San Francisco, CA, United States; ^2^Department of Anesthesia and Perioperative Care, Center for Cerebrovascular Disease, University of California, San Francisco, San Francisco, CA, United States

**Keywords:** perioperative neurocognitive disorder, IL-6, IL-6 trans-signaling, aging, neuroinflammation, Olamkicept, PND, Alzheimer’s disease, POD

## Abstract

For most, staying “mentally sharp” as they age is a very high priority that may be thwarted by the consequences of a postoperative complication unrelated to the disorder which necessitated the surgical intervention. Perioperative neurocognitive disorder (PND) is an overarching term for cognitive impairment in surgical patients, that includes conditions from delirium to dementia, affecting more than 7 million patients annually in the US, and which threatens both functional independence and life. Clinical trials and meta-analyses have identified the association between PNDs and increased perioperative levels of Interleukin-6 (IL-6), a pleiotropic cytokine that is both necessary and sufficient for postoperative memory decline in a preclinical model of PND. Recently, we reported that, in adult male wild-type mice subjected to tibial fracture under general anesthesia, IL-6 trans-signaling in hippocampal CA1 neurons mediates surgery-induced memory impairment. As there are no therapeutic options for preventing or reversing PNDs, patients and their caregivers, as well as the healthcare industry, endure staggering costs. Olamkicept, a highly selective IL-6 trans-signaling blocker has shown to be efficacious and safe in clinical trials involving patients with inflammatory bowel disease, another condition for which IL-6 trans-signaling is the mediating mechanism. Subject to a demonstration that olamkicept is effective in preventing cognitive impairment in vulnerable (aged and Alzheimer’s Disease) preclinical PND models, clinical trials involving aged and/or cognitively impaired surgical patients should be undertaken to study olamkicept’s utility for PNDs.

## Introduction

Inflammation plays an important role in response to surgical injury. The inflammatory response is a defense mechanism that aims to repair the injured tissue and adapt to stress by restoring homeostasis (Medzhitov, 2008). However, when dysregulated, the inflammatory response can lead to a pathologic state and chronic inflammation (Medzhitov, 2008). In 1989 Nishimoto and colleagues published one of the first studies showing a time course of changes in Interleukin-6 (IL-6) levels during the perioperative period (Nishimoto et al., 1989); in a small cohort of 3 patients, IL-6 increased after surgical trauma, reaching peak levels 24 h after surgery (Nishimoto et al., 1989). In 1992, Naito and colleagues reproduced similar results; using a cohort of patients that underwent abdominal surgery, they showed a time course of IL-6, with peak increases observed on postoperative day 1 (Naito et al., 1992). These early studies hinted that IL-6 may play a role in the consequences of the surgery-induced inflammatory response (Zhang et al., 2023). During the last decade, IL-6 has been identified as a biomarker for several neuroinflammatory diseases, including depression and Alzheimer’s disease (Miller et al., 2009; Calsolaro and Edison, 2016; Lyra et al., 2021). This association between neuroinflammation and long-term cognitive impairment suggests that there may be a similar role for IL-6 in the development of cognitive impairment that occurs after surgery, which are known collectively as Perioperative Neurocognitive disorders (PND) (Subramaniyan and Terrando, 2019; Barreto Chang et al., 2022a). We will focus on the role of IL-6 on PNDs and consider whether its signaling mechanism can be a potential therapeutic target.

### Neuroinflammatory response to aseptic trauma in animal models

**Figure 1:**
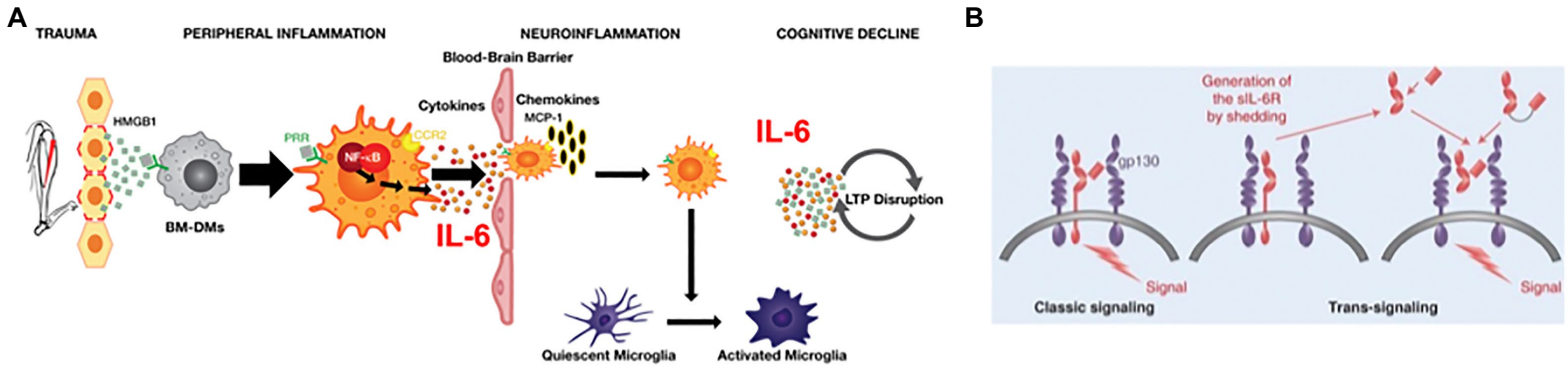
**(A)** Pathogenic model for Perioperative Neurocognitive Disorder in Mice. Peripheral trauma releases the alarmin HMGB1 which binds to pattern recognition receptors (PRRs) on circulating CCR2-expressing bone marrow-derived monocytes (BM-DMs) to transduce peripheral inflammation by translocating the transcription factor NF-kB into the nucleus and upregulating the synthesis and release of pro-inflammatory cytokines. High cytokine levels disrupt the blood brain barrier permitting entry of cytokines and BM-DMs into the hippocampus attracted by the chemokine MCP-1. Resident microglia become activated and, together with BM-DMs, cause further release of cytokines (including IL-6) that signal through transduction pathways to disrupt long-term potentiation (LTP). **(B)** IL-6 signaling: In classic signaling circulating IL-6 binds to membrane-bound receptor, IL-6Ra, resulting in dimerization of the transduction component, gp130, which triggers intracellular signaling. In trans-signaling, the shed ectodomain of IL-6R, sIL-6R, binds to IL-6 and the resulting heteroduplex can trigger intracellular signaling by directly binding to the gp130 dimer in the absence of IL-6R. (Adapted from PMID: 28620096).

The inflammatory response launched by the innate immune system in response to trauma has been systematically studied to identify the specific roles that cytokines and immune cells play in the development of Perioperative Neurocognitive Disorders (PNDs). From the results of these studies, we have generated a pathophysiologic model of PND, depicted in Figure 1A. Systemic inflammation is initiated upon the release from traumatized tissue of damage associated molecular patterns, including high molecular group box protein 1 (HMGB1) (Vacas et al., 2014). HMGB1 binds to pattern-recognition receptors (PRR), including the receptor for advanced glycation end-products (RAGE), on chemotactically attracted circulating bone marrow–derived monocytes (BM-DMs). Activation of RAGE-dependent signaling pathways in BM-DMs translocates cytosolic nuclear factor κB (NF-κB) to the nucleus resulting in the upregulation of proinflammatory cytokine genes, including *Tnf α*, *Il1β*, and *Il6*; within hours of trauma, the cognate proteins of these genes peak in the circulation (Cibelli et al., 2010). Perioperative neutralization of either HMGB1 (Vacas et al., 2014; Terrando et al., 2016) tumor necrosis factor α (TNFα) (Terrando et al., 2010), IL-1β, (Cibelli et al., 2010) or IL-6 (Hu et al., 2018) can block the development of postoperative cognitive decline but may also interfere with healing. Systemic inflammation transforms into neuroinflammation although the mechanisms have not been fully clarified. Despite an intact blood–brain barrier (BBB) that curtails passage of inflammatory mediators and cells, the brain responds to peripheral inflammatory signals through the binding to, and activation of, pro-inflammatory cytokine receptors on either the afferent vagus nerve (Steinberg et al., 2016) or endothelial cells (Salvador et al., 2021) that contribute to the BBB; neuroinflammation then ensues. Additionally, the BBB can be disrupted by high levels of circulating proinflammatory cytokines, which decrease the BBB’s tight junctions (Lopez-Ramirez et al., 2012). Through the permeabilized BBB, circulating CCR2-expressing BM-DMs translocate into the brain parenchyma after trauma (Degos et al., 2013), attracted by the chemokine MCP-1, a ligand for CCR2 receptors which is upregulated by HMGB1 (Vacas et al., 2014). Microglia, the resident brain macrophage, sense the changes induced by both cytokine signaling as well as from the influx of BM-DMs, and become activated (Hovens et al., 2015), resulting in polarization to a proinflammatory phenotype, thereby releasing cytokines including IL-6 (Cibelli et al., 2010), which disrupt synaptic plasticity processes (Terrando et al., 2013) that are associated with learning and memory (Figure 1A; Compton and Bonderman, 1976). Chemokines are also released, which attract more immunocytes, thereby providing a feed-forward loop that perpetuates neuroinflammation (Feng et al., 2017). While neuroinflammation can interrupt synaptic plasticity, neurotransmission, and promote neuroapoptosis, its cellular and molecular constituents may also be a mechanism for regeneration and repair, for example, through release of trophic substances such as brain-derived neurotrophic factor (BDNF) from a reparative microglia phenotype (Zou et al., 2021). Interventions that selectively prevent activation of microglia, e.g., by blocking the Kv1.3 ion channel, can prevent postoperative cognitive decline while leaving intact the peripheral inflammatory response and wound healing (Lai et al., 2020). In the same manner that activation of microglia can result in co-existent proinflammatory or pro-regenerative phenotypes (Paolicelli et al., 2022), astrocytes (another glial cell type) can similarly change morphological (and possible functional) phenotypes, thereby losing trophic regenerative functions (Liddelow et al., 2017). In an aged mouse model of PND, surgery induces astrocytes to express complement 3, a marker previously related to neurotoxicity (Chen et al., 2021). Resolution of inflammation begins soon after initiation and involves both humoral (Chiang and Serhan, 2020) and vagal (Gallowitsch-Puerta and Tracey, 2005) mechanisms. Key inflammatory processes that were noted in animal models are also present in surgical patients. Shortly after surgical incision, circulating HMGB1 levels begin to rise (Saxena et al., 2020) followed by an increase in pro-inflammatory cytokines (Hirsch et al., 2016; Lammers-Lietz et al., 2022), disruption of the BBB (Danielson et al., 2018), infiltration of monocytes into the brain (Berger et al., 2019) and activation of microglia (Forsberg et al., 2017).

### A role for IL-6 in the pathogenesis of PNDs

IL-6 is one of the most congruent biomarkers found in both animal models and clinical studies of PND; yet we lack sufficient knowledge how to best target the neurotoxic effects of this pleiotropic cytokine. IL-6 is the only reported molecule to be both necessary and sufficient to produce the surgical phenotype (Hu et al., 2018); furthermore, in GFAP-IL-6 transgenic mice that overexpress IL-6 in the brain, spatial memory is impaired (Chesworth et al., 2021), possibly due to dysregulated LTP (Hunter and Jones, 2015). Other proinflammatory cytokines, such as TNFα (Terrando et al., 2010) and IL-1β (Cibelli et al., 2010) IL-1β, that are implicated in the development of PNDs, may exert its effects by upregulating *Il6* gene expression (Hunter and Jones, 2015), thereby indirectly contributing to the surgical phenotype. The pleiotropic actions of IL-6, ranging from immune responses to bone healing (i.e., both pro-and anti-inflammatory), are produced by two signaling mechanisms (Uciechowski and Dempke, 2020). “Classic signaling” occurs when IL-6 binds to membrane-bound receptors (IL-6Rα) that are primarily located on hepatocytes, leukocyte subpopulations (including monocytes) and megakaryocytes and transduces its cellular responses through the dimerization of gp130 (Figure 1B; Rose-John, 2018). “Trans-signaling” refers to a process in which IL-6 binds to soluble IL-6 receptors (sIL-6R) that are produced either through proteolytic cleavage of the ectodomain of membrane-bound IL-6Rα by the adamalysin family of metalloproteinases, or by alternative splicing; the IL-6/sIL-6R complex binds directly to a dimer of gp130 and can produce its response in gp130^+^/IL-6Rα-cells (Rose-John, 2021).

### A role for IL-6 trans-signaling in the pathogenesis of PNDs

**Figure 2:**
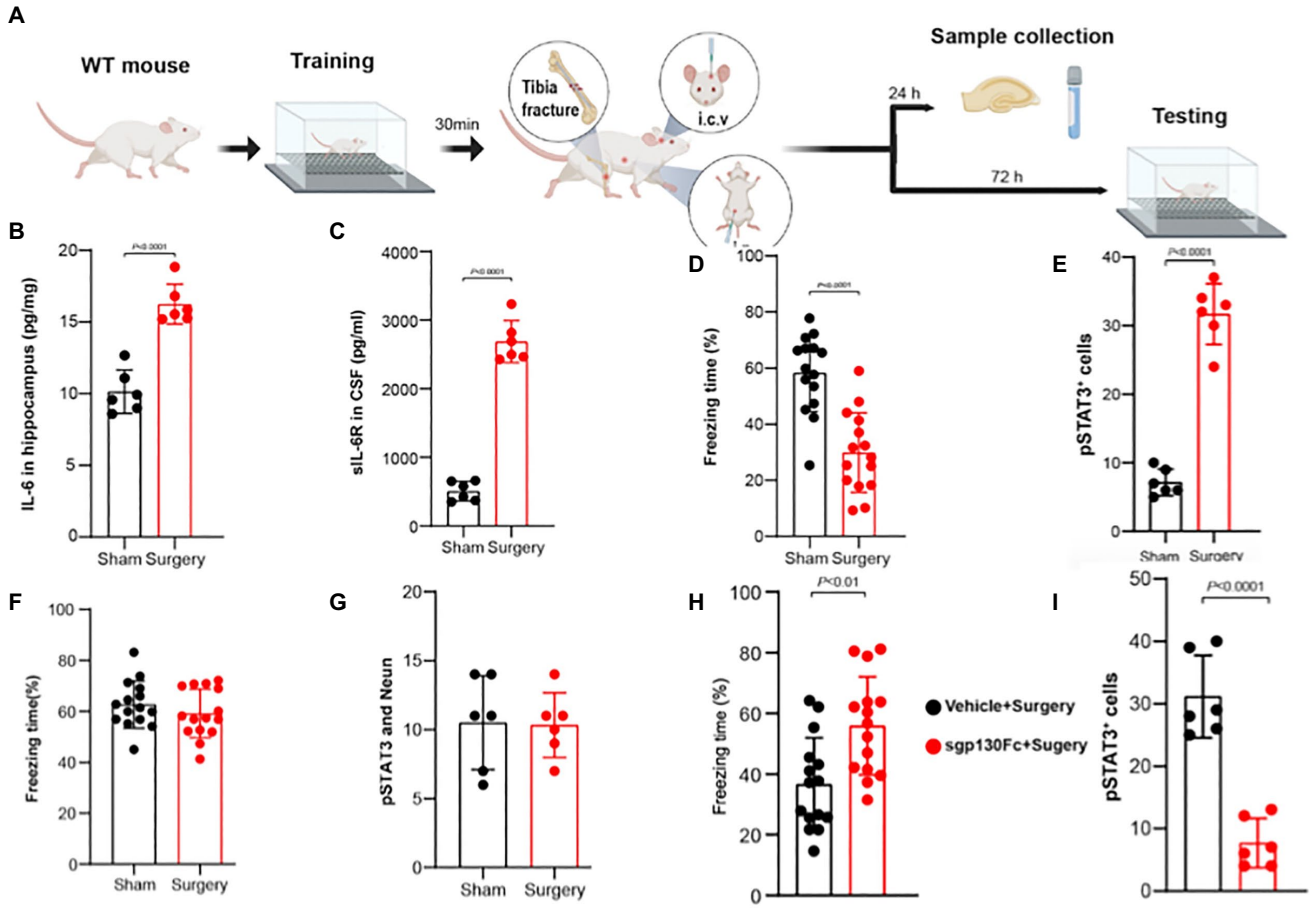
IL-6 trans-signaling in CA1 hippocampal neurons produces postoperative memory impairment. **(A)** Adult male mice underwent training for trace fear-conditioning (TFC) prior to tibia fracture surgery. At 24 h mice were killed and samples harvested; separate cohorts underwent testing in TFC at 72 h. Postoperatively, hippocampal IL-6 levels **(B)** and CSF sIL-6R levels **(C)** rise significantly. **(D)** Freezing time in the TFC declines significantly in the surgical group indicating postoperative memory impairment. **(E)** The number of pSTAT3 ^+^ neurons (counterstained with NeuN) increased significantly after surgery indicating upregulated IL-6 signaling. Following depletion of gp130 in the CA1 region of the hippocampus with direct injection of a viral vector containing a cassette to knockdown gp130 in excitatory neurons, surgery no longer decreases freezing time **(F)** nor increases the number of pSTAT3^+^/Neun^+^ cells **(G)**. Following administration of the selective IL-6 trans-signaling blocker, sgp130Fc, both postoperative freezing time **(H)** and the **(I)** number of pSTAT3 ^+^ neurons return to normal (Adapted from PMID: 36253222).

In a series of genetic and pharmacologic techniques in adult non-vulnerable mice, it was recently reported that IL-6 trans-signaling in hippocampal CA1 neurons is responsible for postoperative memory impairment (Hu et al., 2022); the key findings in this study are depicted in (Figure 2) and are now laid out in further detail. The key components for IL-6 trans-signaling are IL-6 and soluble IL-6 receptors (sIL-6R) that form the heteroduplex required for binding directly to dimerized transduction molecules of sgp130. After aseptic surgery both hippocampal IL-6 (Figure 2B) as well as sIL-6R (Figure 2C) in the CSF are upregulated and are associated with both a decline in memory as depicted in the trace fear-conditioning paradigm (Figure 2D) and an upregulation of phosphorylated signal transducer and activator of transcription 3 (pSTAT3), downstream of gp130, in hippocampal neurons (Figure 2E). When gp130 was downregulated in the hippocampal neurons neither postoperative memory impairment (Figure 2F) nor pSTAT3 upregulation (Figure 2G) occurred indicating that it is in the CA1 hippocampal neurons that the crucial IL-6 trans-signaling occurs to produce PNDs. Pretreatment with i.c.v. sgp130Fc, the selective blocker of IL-6 trans-signaling, prevented postoperative cognitive impairment (Figure 2H) and pSTAT3 upregulation in the hippocampus (Figure 2I). Administration of Hyper IL-6, the selective IL-6 trans-signaling agonist (Peters et al., 1998), upregulated hippocampal pSTAT3 and induced memory impairment in the global absence of IL-6Rs (Hu et al., 2022), an essential component for classic signaling (Rose-John, 2018). Therefore, trans-signaling is the mechanism whereby IL-6 produces postoperative cognitive impairment as suggested for this cytokine’s other neuropathologic effects (Campbell et al., 2014).

### A role for IL-6 in producing enhanced vulnerability to PNDs

According to meta-analyses, the pre-operative risk factors associated with the highest likelihood of developing postoperative delirium (POD) are advanced age (Wu et al., 2021) and existing cognitive impairment including dementia (Cao et al., 2019). Inflammaging refers to a low grade pro-inflammatory state that occurs with advanced age (Franceschi et al., 2000) which may be due to changes in the number and function of innate immune cells, altered expression of PRRs, and its activation by endogenous ligands from cellular damage that results in proinflammatory cytokine secretion (Shaw et al., 2013). The aging brain is characterized by increased expression of genes reflecting activation of microglia and perivascular macrophages, and upregulation of essentially all pathways of the innate immune system (Cribbs et al., 2012). In addition, failed resolution of inflammation and age-related hypovagotonia can contribute to inflammaging (Barantke et al., 2008). Higher circulating levels of IL-6 predict onset of cognitive disability in older persons (Ferrucci et al., 1999) as well as cognitive decline in a 10-yr. longitudinal study (Singh-Manoux et al., 2014). There is a greater likelihood of cognitive impairment in individuals with consistently high or increasing levels IL-6; each doubling of IL-6 changes over 20 yrs. was associated with greater odds of cognitive impairment (Wichmann et al., 2014). Aged mice also have higher levels of IL-6 in the hippocampus especially amongst females (Porcher et al., 2021). Microglia isolated from aged mice have higher expression of IL-6Rα compared to adults; furthermore, expression of hippocampal mRNA for ADAM17, an enzyme responsible for the generation of sIL-6R (required component for IL-6 trans-signaling), is higher in aged mice (Burton et al., 2013). Aged mice have increased priming (MHCII expression) of microglia, likely through IL-6 trans-signaling because there is an increase in sIL-6R in the CSF without a concomitant rise in soluble gp130 (sgp130), the endogenous trans-signaling inhibitor (Garner et al., 2018). More severe and prolonged LPS-induced sickness behavior (including memory impairment) is present in aged mice and is associated with increased expression of hippocampal pSTAT3, both of which can be prevented by i.c.v.-administered sgp130 (Burton and Johnson, 2012).

While the pathognomonic neuropathological features of Alzheimer’s Disease [AD; the most prevalent cause of existing cognitive impairment in surgical patients (Xie and Tanzi, 2006)] are β-amyloid (Aβ) plaques and neurofibrillary tangles from hyperphosphorylated tau protein, neuroinflammation (evidenced by activated microglia/upregulated pro-inflammatory cytokines) is also identified in postmortem brain samples from AD patients (Kinney et al., 2018). In AD, the resolution of inflammation is impaired, resulting in chronic inflammation which can exacerbate AD pathology (Whittington et al., 2017). Aβ activates microglia to phagocytose the plaques (Simard et al., 2006); the phagocytic process is overwhelmed later in the disease (Hickman et al., 2008). When proinflammatory cytokine release from microglia predominates, IL-6 immunoreactivity becomes prominent around Aβ plaques, and IL-6 levels correlate with the degree of dementia (Hampel et al., 2005). Administration of exogenous Aβ, i.c.v., increased circulating levels of IL-6 (Song et al., 2001). Higher levels of IL-6 are also associated with late onset AD (Dursun et al., 2015) and patients with severe AD have higher plasma levels of IL-6 compared to patients with less severe disease or healthy controls (Brosseron et al., 2014). CSF sIL-6R levels are elevated in AD compared to age-matched controls (Delaby et al., 2015). Components of IL-6 trans-signaling, namely IL-6 and sIL-6R, can increase the synthesis of amyloid precursor protein by human neurons (Ringheim et al., 1998). IL-6 can increase CDK5 activity to hyperphosphorylate tau epitopes. Inhibition of IL-6 trans-signaling can prevent the behavioral and pathophysiologic phenotype (Escrig et al., 2019) as well as metabolic abnormalities (Escrig et al., 2020) in AD-like mouse models. In summary, IL-6 is positioned to mediate vulnerability to postoperative cognitive impairment in aging and AD and possibly to drive longer term neurodegenerative changes that can further exacerbate postoperative cognitive decline.

### IL-6 and perioperative neurocognitive disorders

For older patients, PND is the most common postsurgical complication (Berger et al., 2018). Strong preoperative risk factors include age, baseline cognitive impairment, and type of surgery (Barreto Chang et al., 2022b). In the past decade, multiple studies have been performed to evaluate the role of preoperative inflammatory mediators and their relationship to postoperative delirium. Noah and colleagues performed a meta-analysis that evaluated preoperative blood levels of inflammatory mediators, including IL-6 (Noah et al., 2021); six studies that had preoperative IL-6 and measures of POD were included. They reported that preoperative IL-6 was significantly higher in participants who developed POD compared with those who did not (Noah et al., 2021). Altogether, the results provide evidence for an association between preoperative elevated IL-6 and POD, suggesting that a pro-inflammatory state might increase the risk of developing POD.

Additional studies evaluating the role of IL-6 not only before surgery but also during the postoperative period were done by Liu and colleagues. In this meta-analysis, the authors included 7 studies investigating preoperative IL-6 levels and 6 studies that included postoperative IL-6 levels. They found that elevated preoperative and postoperative IL-6 levels are associated with POD (Liu et al., 2018). They also investigated if there was an association with postoperative NCD (referred to as postoperative cognitive dysfunction, POCD). In this case, elevated preoperative levels of IL-6 (16 studies) were not associated with postoperative NCD, but elevated IL-6 after the surgery (17 studies) was associated with postoperative NCD (Liu et al., 2018). These differences may be due to different mechanisms associated with POD but not necessary with postoperative NCD. Although some patients that develop POD also develop postoperative NCD, not all do, pointing to diverse mechanisms that can lead to the different PNDs. It is important to note significant heterogeneity between the studies (Liu et al., 2018). Additional limitations include the timing of the sampling, sample processing, and heterogeneity among the groups studied. These differences can influence the IL-6 concentrations detected which can lead to discrepancies among studies. Altogether, the results provide evidence for an association between elevated IL-6 and PNDs.

### Beyond IL-6, role of sIL-6R, and trans-signaling pathway

IL-6 exerts its biological functions *via* both the classic and the trans-signaling pathways (Garbers et al., 2018). However, some studies suggest that the trans-signaling pathway, rather than the classic pathway, is involved in the development of PND (Hu et al., 2022; Zhang et al., 2023). In the first study investigating the association of sIL-6R and POD in humans, Neerland and colleagues (Neerland et al., 2016) prospectively studied a cohort of 126 patients that underwent major lower limb surgery. They found that there was a trend to higher preoperative CSF levels of sIL-6R in participants that had baseline cognitive impairment and developed POD compared to those that did not develop POD (*p* = 0.06) (Neerland et al., 2016). A second study by Zhang and colleagues explored IL-6 and sIL-6 and their relationship to POD, evaluating different time points before and after surgery (Zhang et al., 2023). They classified the patients based on two trajectories: stable lower and fluctuating higher levels. They found that fluctuating higher IL-6 and sIL-6R levels were significantly associated with higher risk of POD supporting a role for the trans-signaling pathway (Zhang et al., 2023).

### Il-6 trans-signaling as a therapeutic target for POD

Given the new evidence of the role of the IL-6 trans-signaling pathway in the development of POD, it presents an opportunity to explore the therapeutic targets represented by this pathway. Targeting only the trans-signaling pathway would avoid blockade of the important physiologic functions mediated by the classic IL-6 signaling pathway (Garbers et al., 2018). Olamkicept (sgp130Fc), an inhibitor of the trans-signaling pathway, is a potential therapeutic intervention and has been recently used in clinical trials on patients with inflammatory bowel disease (IBD) (Schreiber et al., 2021). For IBD it is thought that upregulation of the proinflammatory activity of IL-6 mediated by the trans-signaling pathway contributes to the pathogenesis. For this reason, the trans-signaling inhibitor is currently being studied in two phase II clinical trials for IBD (Schreiber et al., 2021). Olamkicept, exclusively blocks IL-6 proinflammatory trans-signaling and has shown efficacy in preclinical models of IBD, without signs of immunosuppression (Schreiber et al., 2021). In humans, Olamkicept was well tolerated and induced clinical response in 44% and clinical remission in 19% of IBD patients and decreased pSTAT3 signaling in the mucosa of inflamed bowel (Schreiber et al., 2021). Adverse events have been studied on animals, which suggest that treatment with olamkicept does not cause immune suppression. However, the small number of patients included in the current human studies (n = 10, that completed the clinical trial), limits the evaluation of adverse events. The investigators recognized this limitation and are currently conducting a large placebo-controlled trial (NCT03235752) to further investigate whether gp130 trans-signaling blockade does not cause any immune suppression in humans. In a second Phase II placebo-controlled double-blind trial involving 91 patients with moderate to severe ulcerative colitis, a dose of olamkicept of 600 mg biweekly resulted in significant (*p* = 0.032) clinical improvement in 58.6% patients compared to 34.5% of placebo-treated patients; full clinical remission at 12 weeks occurred in 20.7% of patients vs. 0% in the placebo group with no difference in treatment emergent adverse events (Chen et al., 2021).

Olamkicept is a novel treatment for conditions in which the IL-6 trans-signaling pathway is upregulated and may be repurposed for PNDs in the future. For these putative clinical trials, different time points during the perioperative period should be considered. Patients with high preoperative levels of IL-6 could potentially benefit from prophylaxis with Olamkicept. In addition, there is some evidence that postoperative PND is associated with elevated postoperative levels of IL-6. However, the association between the trans-signaling pathway and the development of postoperative PND has not yet been explored in humans. Thus, additional studies characterizing the mechanisms and the role of IL-6 and sIL-6R as biomarkers for PNDs are needed to appropriately determine the time points at which the intervention may be most effective.

The patient population is another important consideration. Older adults are at the highest risk for POD and postoperative PND (Monk et al., 2008; Barreto Chang et al., 2022a). Besides age, baseline cognitive impairment is a strong predictor of PNDs. Early baseline cognitive assessment can also help identify those patients with higher vulnerability. Furthermore, the type of surgery and complexity of the case can increase the risk for POD (Barreto Chang et al., 2022b). Gathering the individual risk factors, baseline inflammatory biomarkers, and cognitive assessment could be the first considerations to explore which patients may benefit most from therapies targeting IL-6 trans-signaling.

## Conclusion

Recent preclinical studies from a single investigator group have established an association between IL-6 trans-signaling in CA1 hippocampal neurons and the development of PNDs; further corroborative studies are needed. With the effective and safe intervention of olamkicept, a selective IL-6 trans-signaling blocker, for IBD, another disease process for which this signaling mechanism is involved, the stage is set to re-purpose this pharmaceutical preparation for clinical trials in patients at high risk for the development of PNDs. Furthermore, the efficacy can also be considered for “medical” causes of delirium, including that which occurs in the setting of COVID-19.

## Author contributions

OBC and MM conceptualized and wrote the manuscript. All authors contributed to the article and approved the submitted version.

## Funding

OBC received support from the UCSF School of Medicine Irene Perstein Award.

## Conflict of interest

The authors declare that the research was conducted in the absence of any commercial or financial relationships that could be construed as a potential conflict of interest.

## Publisher’s note

All claims expressed in this article are solely those of the authors and do not necessarily represent those of their affiliated organizations, or those of the publisher, the editors and the reviewers. Any product that may be evaluated in this article, or claim that may be made by its manufacturer, is not guaranteed or endorsed by the publisher.
